# Yellow Fever at Staten Island, near New York

**Published:** 1848-10

**Authors:** 


*Yellow Fever at Staten Island, near New York.*—A form of disease has recently made its appearance near the Quarantine grounds, at Staten Island, opposite New York, which was regarded by the Health Physician, and others, as *yellow fever.* We give below portions of the Report by the Health Physician to the Board of Health on the subject, and the action of the Board thereupon, by which it will be perceived, that its origin is attributed to vessels from New Orleans which had cases of yellow fever on board, and that the landing of vessels at the Quarantine ground, and cessation of intercourse by fences, &c., are interdicted. We have been assured by Dr. Stone, of New Orleans, who examined the cases, and whose ability to recognize the disease may be confidently relied upon, that its identity with the yellow fever of New Orleans is unquestionable. We learn, also, from a private source, that the characteristic post mortem appearances, as described by Louis in his essay on the yellow fever at Gibralter, have been discovered in all the fatal cases that have been examined. Some of the physicians of New York, as is intimated, however, deny that it is yellow fever. The question of its importation, also, gives rise to difference of opinion, involving, as it does, the long mooted question of contagion, a question the negative of which we had supposed had at length been pretty conclusively settled. We trust that the Health offlcer will consider it as falling within his duties to publish a full report of all the facts appertaining to the occurrence of the disease, its progress, etc., for the satisfaction of the profession at large.

"At a meeting of the Board of Health, Aug. 30, a letter to the Mayor from the Health Officer, Dr. Alexander B. Whiting, was read, the principal facts of which are contained in the following extracts."

"I deem it my duty to inform you that there exists at this place, and in the villages of Tompkinsville and Stapleton, adjoining the quarantine grounds, a disease that has within a day or two past assumed the character of a malignant yellow fever. Certain symptoms and a mild form of the same disease have prevailed for a period of ten or twelve days, but not sufficiently decided to justify its designation as yellow fever by all the physicians in and about this neighborhood; but within a few days a number of cases have occurred with such definite symptoms as to leave their nature established.

"The first cases occurred in the quarantine grounds, among the boatmen employed in the health officer's barge and those of the revenue barge, and one or two engaged on the steamboat dock, contiguous to this place. Subsequently the cases occurred among persons living near the shore, in a district extending about a mile south, and a quarter of a mile in shore.

"The number of cases thus far has been about fifty. Of these the malignant cases, numbering twelve, commenced on Wednesday, the 23d of August, and have been occurring daily since. Six deaths have occurred from among these, with all the symptoms of the disease so decided as to leave no doubt in the minds of the medical gentleman who have seen them concerning its nature.

"For the causes of the disease I think we must look to vessels from New Orleans, lying at quarantine, having had yellow fever on board. These are the barque Edgar and ships George Hews and Callander. They have been removed as far as possible from shore; sufficiently to obviate the danger from them."

A select committee of five was immediately appointed, to act in connection with the Health Commissioners, to examine into the origin and existence of the disease, and to suggest such means as they might think necessary for the Board to adopt. All communication from the city with the infected district was also prohibited. On the following morning the committee reported, at an adjourned meeting, as follows:—

"That the disease in question had its origin in the present locality to the presence of the vessels at quarantine, as named in the communication of the health officer to the board, at their meeting of yesterday. That these vessels, to wit, the George Hews, Edgar and Callander, had each arrived at the quarantine station from New Orleans, which port they left since the 26th of July. That the former had seventeen cases during the passage, of which seven terminated fatally, the Edgar lost three cases and the Callander one. That the above vessels on their arrival at our port anchored at the usual anchorage ground, which is about a quarter of a mile from shore, and that the prevailing wind during the time of their lying there had been from the north east, and blowing directly on the shore where the disease prevails.

"That the first cases of the disease were, as stated in the communication of the health officer, among the persons employed in and about the quarantine ground—and that subsequently it appeared on the shore adjacent to these grounds, in the form of a mild ordinary fever, which afterwards assumed a malignant character, and was, after a full investigation by the physicians, pronounced yellow fever. The disease, as it now exists, is confined to the shore of the island—not extending inland more than half a mile—and the length of the district affected being rather more than a mile, which district includes the Quarantine and Stapleton ferry landings.

"The number of persons on shore affected by the disease, since its appearance about a fortnight since, in its mildest form, is about 34 cases, and that of the malignant, 16—the first of which appeared on the 23d instant; and of these latter, seven have terminated fatally.

"The health officer has had the above-named vessels removed to an anchorage ground in the lower bay, more remote from the shore, and where little or no population is to be found, and ordered a large distribution of the usual disinfecting agents along the coast of the island, as far as the same is known to be infected.

"Your committee recommend a continuance in force of the resolution, as passed by the board, requiring a cessation of intercourse by means of the usual ferry or other vessels, and they further recommend to their fellow citizens, and require from them, a strict observance of these and such other sanitory regulations as the board may feel themselves called upon to enforce."

The report was accepted, and a resolution adopted that all communication between the city and Vanderbilt's landing, on Staten Island, be for the present suspended."

